# Foundations of form and function: A synthesis‐based curriculum for introductory‐level organismal biology

**DOI:** 10.1002/ece3.8315

**Published:** 2021-11-09

**Authors:** Laura N. Kloepper, Vanessa K Hilliard Young

**Affiliations:** ^1^ Department of Biology Saint Mary’s College Notre Dame Indiana USA

**Keywords:** course design, introductory biology, pedagogy, remote learning

## Abstract

First‐year majors organismal biology courses are frequently taught as survey courses that promote memorization rather than synthesis of biological concepts. To address the shortcomings of this approach, we redesigned the organismal portion of our introductory biology curriculum to create a “Foundations of Form and Function” course. Foundations of Form and Function introduces different organismal forms and focuses on the relationship between those forms and the execution of key physiological functions. Goals of our new course include the following: developing student recognition of common characteristics that unite living organisms as well as features that distinguish taxonomic groups, facilitating student understanding of how organisms accomplish similar functions through different forms, and reinforcing course themes with independent student research. In this paper, we describe course learning outcomes, organization, content, assessment, and laboratory activities. We also present student perspectives and outcomes of our course design based on data from four years of student evaluations. Finally, we explain how we modified our course to meet remote learning and social‐distancing challenges presented by the COVID‐19 pandemic in 2020 and 2021.

## Graphical abstract

We redesigned the organismal portion of our introductory biology curriculum to create a “Foundations of Form and Function” course. Goals of our new course include the following: developing student recognition of common characteristics that unite living organisms as well as features that distinguish taxonomic groups, facilitating student understanding of how organisms accomplish similar functions through different forms, and reinforcing course themes with independent student research. In this paper, we describe course learning outcomes, organization, content, assessment, and laboratory activities; we also present student perspectives and outcomes of our course design based on data from four years of student evaluations.

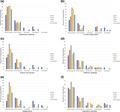

## 1 INTRODUCTION

Across colleges and universities, first‐year majors biology courses are often taught as survey courses, covering a wide range of topics spanning the breadth of biology (Soto & Everhart, 2016). For organismal biology, a core component of nearly every first‐year biology curriculum, this is often taught as a brief introduction to the key characteristics of prokaryotes, protists, plants, and fungi, followed by a “march through the phyla” approach for the animal kingdom and ending with an introduction to anatomy and physiology that focuses heavily on systems in humans (Mason et al., 2020; Starr et al., 2018; Urry et al., 2017). Although these courses are helpful in introducing students to the varied topics they will need for different biology concentrations or careers, they are often taught in a manner that leads to memorization of facts rather than a synthesis of biological concepts, which can limit how students apply and analyze course content (Anderson et al., 2000). As a result, students in traditional survey courses may perceive vocabulary to carry more importance than scientific concepts (Rigden & Tobias, 1991; Tobias, 1990, 1992) and often fail to connect concepts within the biological sciences. Furthermore, the organization of textbook material, and subsequently lecture content, does not lend itself to students developing a functional understanding of organismal biology, as many undergraduate students have not yet developed the skills necessary to organize course information in meaningful ways (Ambrose et al., 2010). It is important, then, that instructors help guide students through organization and integration of concepts that allow students to understand functional relationships across multiple systems and organisms.

Motivated by the limitations of traditional survey courses, we aimed to redesign the organismal portion of our introductory biology curriculum. Specifically, we renamed our course “Foundations of Form and Function” and restructured the curriculum to focus on how specific physiological functions are achieved using different forms that span organismal diversity. We had three goals with our new course: (1) help students understand the characteristics that unite living organisms and traits that are unique to groups of taxa, (2) help students understand how living organisms achieve similar functions through different forms, and (3) reinforce overall course themes with laboratory investigations and independent research. Below, we provide an overview of the course including learning outcomes, lecture organization, content, assessment, and laboratory activities. We also provide data from four years of student evaluations and explain how we modified our course in 2020 and 2021 due to remote and socially distanced learning scenarios resulting from the COVID‐19 pandemic.

## 2 PEDAGOGICAL OVERVIEW

### 2.1 Course design and learning outcomes

Our Foundations of Form and Function course (“F&F”) is the final module of a four‐part Introductory Biology sequence for Biology majors at Saint Mary's College (Notre Dame, IN, USA). This seven‐week course includes both lecture and a six‐week laboratory component, totaling six contact hours per week, most weeks. F&F covers the diversity of life of Earth, with specific emphasis on the relationship between form and function as it pertains to structure, organization, and movement; nutrients and feeding; transport and gas exchange; reproduction; and sensory systems (Table 1). As an introductory‐level science course, F&F has learning objectives that align with the College's liberal arts curriculum: at the conclusion of the course, students should be able to use scientific methods to investigate questions in the natural sciences; demonstrate understanding of the principles that drive natural phenomena; and identify and evaluate critical issues that they face as citizens of the world. Additional learning objectives specific to F&F include establishing a foundation for understanding biological diversity, developing familiarity with relationships between form and function as they pertain to organismal functions, and advancing research skills and scientific literacy.

**TABLE 1:** Course topics and associated textbook chapters (Mason et al., 2020)

Topic	Text Chapter
Introduction to Diversity	1, 4.2–4.3
Prokaryotes, Protists, and Fungi	27, 28, 31
Introduction to Plants	29, 30
Introduction to Animals	32, 33, 34
Structure, Organization, and Movement	35, 41, 45
Nutrients and Feeding	7.1, 8.1, 37, 46
Transport and Gas Exchange	36, 47, 48
Reproduction	40, 51, 53
Sensory Systems	39, 43

#### 2.1.1 Lectures and content

The lecture component of the course is structured such that students are introduced to organismal diversity and acquire an understanding of key similarities and differences among broad taxonomic groups (Table 1). The course begins with a brief overview of diversity and evolution. We then explore general characteristics of prokaryotes, protists, and fungi, highlighting common and distinguishing features among representatives of these groups, followed by an introduction to plants and animals.

Once broad groups of organisms have been introduced and students are familiar with their key features, we begin developing students’ functional understanding of organismal form and function. F&F focuses on five key organismal functions: structure, organization, and movement; nutrients and feeding; transport and gas exchange; reproduction; and sensory systems. Lectures explore the diversity of form across broad organismal groups and the relationship between structure and the execution of a given physiological function. For example, in the nutrients and feeding lecture, we discuss photosynthesis, the role of nitrogen‐fixing bacteria in plant nutrition, and modifications that allow plant carnivory in low‐nitrogen environments. We also introduce several types of animal feeding (e.g., filter feeding, fluid feeding) and highlight differences in form that accommodate these feeding strategies (e.g., mosquitos have a long, sharp proboscis for piercing tissues to feed on blood). Finally, we wrap up the lecture with an exploration of the vertebrate digestive system. In this part of the lecture, we discuss similarities in form across phyla, such as increased surface area for absorption in both plant roots and the small intestine. We also highlight major differences in form across vertebrate groups (e.g., gizzards in birds; diverse tooth structures in mammals) and how these differences in form accomplish specific functions (e.g., mechanical digestion of food). Lectures are punctuated with interactive elements to encourage student engagement with the material, help with synthesis of course content, and practice scientific literacy skills. For example, some lectures include graphs related to the lecture topic, and students are invited to interpret and explain these graphs to their peers as part of the lecture. Multiple‐choice questions are also incorporated into several lectures, offering the class a chance to pause, reflect on earlier content, and collaborate to determine the correct response. Lectures also conclude with synthesis questions for students to work through independently to help them review and organize content in meaningful ways. For instance, the nutrients and feeding lectures conclude with the following synthesis questions:

1. Why is feeding/acquiring nutrients important for all life?
2. How is feeding/acquiring nutrients similar and different across broad groups of life?

#### 2.1.2 Quizzes

Four quizzes are given during lecture in this class. Quizzes are composed of multiple‐choice questions that reinforce key concepts from lecture and assigned textbook readings. Quizzes are given via Blackboard Learning Management System (Blackboard LMS), and students are able to review quiz questions once all student attempts have been graded. Quizzes are designed to familiarize students with multiple‐choice question format and style in preparation for course examinations.

#### 2.1.3 Homework

Students complete five homework assignments for the lecture portion of F&F. In 2018, broad‐scale synthesis questions were provided to students to work through independently, but this work was not submitted for credit nor included as part of the course assessment. In 2019, these questions were adapted as for‐credit homework assignments; however, students struggled to synthesize the breadth of information covered in lecture. Based on student feedback (see Section 3—Student Perceptions and Outcomes), homework assignments were revised after 2019. In 2020 and 2021, assignments consisted of eight short answer questions that were designed to guide students through the process of synthesizing large amounts of information from lectures and textbook readings and discourage rote memorization (Box 1). By including more questions on the assignment, instructors were better able to highlight core principles that students should focus on. Homework assignments were completed outside of class time and submitted electronically via Blackboard LMS. Assignments were graded based on effort and completion. If a student demonstrated reasonable effort in composing their responses, and all questions were attempted, full credit was granted. To expedite feedback on written assignments, homework keys were made available using the Adaptive Release feature in Blackboard LMS after the assignment deadline to all students who submitted an assignment attempt. This strategy allows students to check their responses against instructor expectations and correct their notes, while still preserving the integrity of the assignment (i.e., releasing the key after the deadline rather than immediately after submission prevents the key from circulating among the class before everyone has completed the homework).

BOX 1 **Sample questions from Foundations of Form and Function homework assignments (2020–2021)**

1. Annelids, arthropods and chordates all share one characteristic. What is this and why is this important?
2. Animals and plants are very different organisms, yet they do share some traits. What are the traits animals and plants share (that we have learned about thus far)?
3. Structure and movement is important for all life. In class, we covered structure and movement in plants and across a broad range of animal taxa. Give specific examples from the following groups of organisms: plants, invertebrates (choose one group) and vertebrates. For each example, explain the FORM (including descriptions of the anatomical structures) and FUNCTION (how the anatomical structures "work") that allow each organism to have structure and movement.
4. Explain how transport is the same across all levels of living organisms (prokaryotes through animals).

#### 2.1.4 Examinations

F&F includes two examinations and a comprehensive final examination at the conclusion of the course. Examinations consist of multiple‐choice and short answer/essay questions that match the scope and style of questions on quizzes and homework assignments. Examinations contain questions that test a student's ability to recall facts and basic concepts, as well as higher level Bloom's taxonomy questions that allow students to apply core concepts, analyze data, and evaluate novel scenarios (Bloom et al., 1956).

#### 2.1.5 Recitation hour

In addition to lecture and laboratory times, F&F includes a weekly one‐hour Recitation Hour on Friday afternoons. Recitation hour attendance is strongly recommended to students, but is not required. Furthermore, the day and time for the recitation hour is included in the registrar's course schedule, so as to limit scheduling conflicts that might prevent student participation in recitation hours during the course. Recitation hours function as an additional development opportunity for students, as well as a time for students to interact with peers and the lecture instructor. Early in the course, recitation hours provide a structured time for the instructor to discuss study habits, answer questions about the course, and offer advice for students to help them successfully reach their personal goals for the class and in the major. Recitation hours also provide a designated review time prior to examinations, during which students can ask questions about course material and/or examination format and expectations. On weeks without a predetermined recitation hour topic, recitation hour serves as an additional “student hour” (i.e., office hours), during which students can meet with the lecture instructor to discuss course performance, clarify concepts, review graded materials, etc.

### 2.2 Laboratory design and independent project

The core of biology as a discipline is investigative research, or the ability to ask questions about biological systems and seek answers. The laboratory component of this course includes the following objectives: exposure to a breadth of scientific methods and techniques, collaboration, forming and testing of a hypothesis, experimental design, data collection and statistical analysis, reading and citing primary literature, oral communication, and scientific writing. Through instructor‐designed exercises and independent research, students develop these skills while building on their knowledge of form and function.

The laboratory section is designed to last six weeks. During the first three weeks, students work in groups, rotating between stations and completing instructor‐created exercises (Table 2). Prior to laboratory, students complete a prelaboratory assignment that is focused on statistical analysis (Week 1), interpreting scientific figures from papers authored by their professors (Kloepper & Bentley, 2017; Young & Gifford, 2013; Week 2), or scientific writing and peer review (Week 3). Each week's hands‐on stations are designed to represent a diversity of organisms and forms associated with executing functions related to feeding (Week 1), locomotion (Week 2), or responding to stimuli (Week 3). While at each station, students collect data with experimentation. Then, in the postlaboratory assignment, students plot and interpret their data and answer open‐ended questions designed to promote inquiry and spark ideas for independent experimentation.

**TABLE 2:** Laboratory topics and exercises

Week	Topic	Prelaboratory	Station 1	Station 2	Station 3
1	Feeding	Statistical Analysis	Photosynthesis (leaf disk assay)	Mammalian Jaw Morphology	Zebrafish Feeding Behavior (video station)
2	Locomotion	Describing Scientific Figures	Maple Leaf Dispersal	Planaria Locomotion	Zebrafish Locomotion (video station)
3	Response to Stimuli	Scientific Writing and Peer Review	*Vorticella* Habituation (video station)	Isopod Tonic Immobility	*Betta splendens* Territorial Behavior

#### 2.2.1 Week 1: Feeding

In the first week of the laboratory, students investigate feeding behavior in plants, mammals, and fish. At Station 1, students investigate how different levels of carbon dioxide affect the rate of photosynthesis using the spinach leaf assay (College Board, 2012). At Station 2, students calculate the lever ratio of several mammalian jaws to predict the corresponding feeding behavior of each species. The final station uses pre‐recorded video data for students to test whether food‐deprived zebrafish consume food faster than satiated zebrafish. Students are also provided scientific papers to supplement each station (Greaves, 1985; Kirschbaum, 2004; Lawrence et al., 2012).

#### 2.2.2 Week 2: Locomotion

The second week of the laboratory introduces students to locomotion in plants, flatworms, and fish. At Station 1, students investigate how length and width of maple seeds affect the horizontal dispersal distance when dropped from a height of approximately ten meters. Students investigate planaria locomotion at Station 2 and test whether water temperature affects righting response time in planaria after being disturbed with a water current. At Station 3, students use pre‐recorded video data to test whether ethanol exposure affects zebrafish locomotor response to a simulated predator. Supplemental papers for Week 2 include Guries and Nordheim (1984), Claussen et al. (2003), and Gerlai et al. (2006).

#### 2.2.3 Week 3: Response to stimuli

During Week 3, students investigate response to stimuli in ciliates, arthropods, and fish. Station 1 uses pre‐recorded video data for students to test whether *Vorticella* habituate to a disturbing and repeated stimulus, with Patterson (1973) as supplemental reading. At Station 2, students investigate how stimulus type (pinch vs. drop) affects tonic immobility duration in isopods (Quadros et al., 2012). At the final station, students investigate whether the color of a fish model affects the territorial response in male *Betta splendens* and use Doutrelant et al. (2001) as additional reading.

#### 2.2.4 Postlaboratory assignments

Each week, the postlaboratory assignment asks students to answer specific questions about form and function at each station, create graphs of their results across all stations, and choose one station for hypothesis testing via appropriate statistical analysis. Students also answer a synthesis question “Compare and contrast the [feeding/locomotion/response to stimuli] behavior of [the different organisms tested across all three stations]. How are these behaviors alike? How are they different?” Finally, students write a short paragraph describing how they would design an experiment of their choosing to further investigate feeding/locomotion/response to stimuli behavior in either of the model organisms used at the stations. These questions are designed to prepare students for the independent research phase of the course.

#### 2.2.5 Weeks 4–6: Independent research

During weeks four through six of the course, students work in pairs to design and complete an independent research project ending with a final laboratory report and conference‐style oral presentation. Students are encouraged to develop research questions related to topics they have explored in laboratory in weeks 1–3, using organisms already available in the laboratory, but are not provided with a list of hypotheses or research questions from which to choose. Such course‐based undergraduate research experiences (CUREs) can improve accessibility of research, enhance student learning, and increase student retention (Bangera & Brownell, 2014; Brownell et al., 2015; Rodenbusch et al., 2016). At the start of Week 4, students submit three experimental design options and hypotheses to their instructor and, based on instructor feedback, select one for their final project. Students conduct their independent experiment outside of laboratory time and receive instructor assistance on logistics, data collection, and statistical analysis during their scheduled laboratory time. During Week 5, students also have the opportunity to workshop portions of their laboratory report with their instructor and receive peer feedback on their writing. The final week (Week 6) of the laboratory ends with students presenting their work to the class in a conference‐style slideshow presentation and submitting a formal laboratory report on their project.

## 3 STUDENT PERCEPTIONS AND OUTCOMES

**FIGURE 1:**
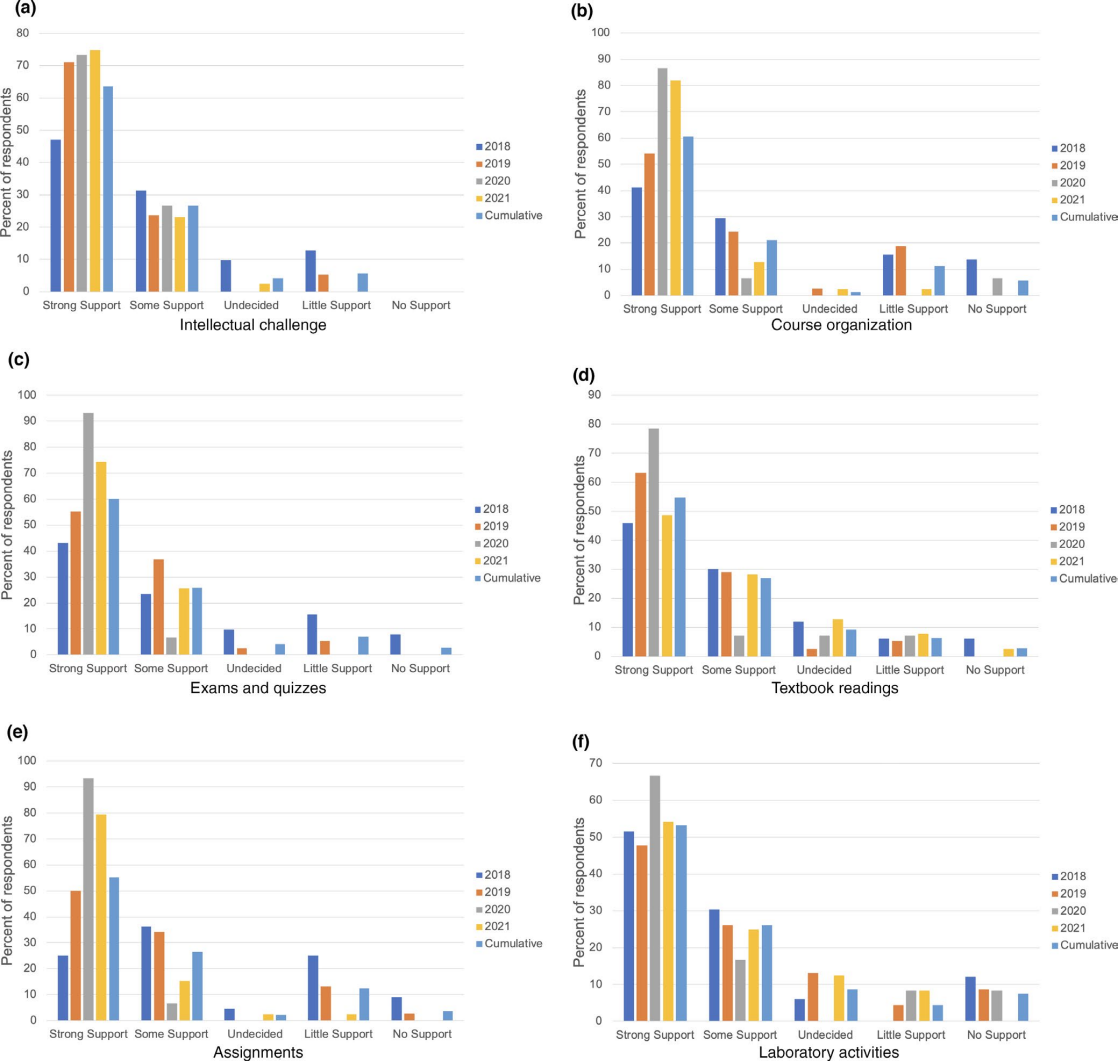
Voluntary student response data from 2018 to 2021 indicating the level of support with which course components supported each student's learning process. (a) Course intellectual challenge, (b) course organization, (c) exams and quizzes, (d) textbook readings, (e) assignments, (f) laboratory activities

To evaluate the student perceptions and outcomes from the course, we compiled data from anonymous, voluntary online evaluations students completed at the end of the course from 2018 to 2021. The overall student response was 49.4% (*n* = 143 students) over the four‐year assessment period. An overwhelming majority of students indicated that course intellectual challenge, organization, examinations and quizzes, textbook readings, assignments, and laboratory activities provide strong or some support to their learning process (Figure 1). Across all four years, course intellectual challenge, organization, and examinations and quizzes provided the strongest support to student learning (Figure 1a–c, Box 2).

BOX 2 **Selected qualitative comments from student course evaluations for Foundations of Form and Function from spring 2018 to spring 2021.**

Intellectual Challenge and Organization:

- I liked how organized the class was; it was very structured.
- The intellectual challenge was very beneficial in setting myself up to work hard to learn the material.
- The organization was most helpful. She (the instructor) had the syllabus ready beforehand and what material we would discuss. I liked how she (the instructor) stated what quiz or exam would have what lecture material.
- The class organization was most supportive in my learning process. The attached powerpoints made it easy to follow along and take crucial notes during lecture.

Exams and Quizzes:

- The quizzes were very helpful because they set the standards for what types of questions were going to be on the exams.
- The quizzes were helpful in preparing for exams, along with the synthesis questions in the lecture powerpoint slides.
- I really liked quizzes because they gave me a sense of how much I knew and helped me study for exams.

Textbook Readings:

- It was challenging to pull parts from the textbook that were helpful but also focused on the correct content of study.
- The textbook was rendered useless. I and many other students continuously felt it was useless. Lecture had all the material needed without the extreme textbook fluff and detail. There may have been students who bought it (a VERY expensive textbook) knowing it was required, but never needed to touch it.
- Her (The instructor's) notes are greatly appreciated because sometimes the textbook is a bit dense. She (The instructor) is very good at answering questions and simplifying the textbook a bit.
- The textbook is a really difficult reference in this class because it's either almost always too specific or too broad.

Assignments (2018–2019):

- I wish there were assignments besides the reading quizzes.
- I would have preferred in‐class graded assignments to serve as a progress check. The quizzes, while great for pre‐lecture, did not give students an idea of how much they knew before the test.
- Assignments in lecture did not support my learning at all because there were none. As much as I dislike homework, it really does help me prepare for tests and quizzes. Given that this is the first year for these short foundations courses, I believe that makes assignments the much more important because it gives you a feel for how your professor will ask you questions on exams.
- I found the assignments to be unhelpful in general.
- I thought the expectations for the homework were confusing.

Assignments (2020–2021):

- Assignments most supported my learning process because the homework was a good check on my information so I could do better on my quizzes and tests.
- Homework assignments supported my learning process the most because it allowed me to put what I learned to practice so that I retain the knowledge.
- The homework also helped pull everything together and prepare for the free‐response questions on the exams.
- The homework assignments were great to synthesize information after each power point. They weren't too long and they felt like a good amount of challenge.
- The homework directly correlated with the exams which made studying a lot easier. The homework was very straightforward and easy to understand, yet difficult to answer. I mean that as a compliment, not a criticism. It forced me to look for the answers rather than googling or guessing on multiple choice.
- The synthesis homework assignments and the synthesis questions were a great help when studying for the exam. These elements were helpful because they encouraged us to find the similarities and differences among the organisms and overall ideas we were learning, rather than just being multiple choice questions we could easily see what the answer was.

Laboratory Activities:

- Lab helped to understand how different organisms are related in different respects
- The element that most supported my learning process was the lab and experiential learning aspect. Conducting experiments and seeing the individual characteristics of different organisms being tested helped me most to understand the differences.
- Observing the animals in lab was very beneficial to understanding lecture.
- The lab most supported my learning process because it allowed me to gain hands on experience. I could see what we were learning being applied to the real world and it helped me to better understand the material.

Course evaluation feedback from 2018 and 2019 indicated that students found assignments less than effective in developing their understanding of course material (Figure 1e; Box 2). In response to student feedback, assignments for F&F were revised between 2019 and 2020. After the change in structure for the course assignments, students indicated a substantial improvement in the effectiveness of assignments in promoting student learning (Figure 1e). Qualitative comments from 2020 and 2021 evaluations indicate that students found homework assignments valuable in helping them synthesize key information from lecture and readings, apply concepts learned in class, and prepare for examinations (Box 2).

For nearly every course component, the percent of students responding that course elements provided strong support for learning occurred during 2020, which was also the year the lecture and laboratory was completely remote and virtual due to the COVID‐19 pandemic. With the challenges of remote learning (elaborated further in Section 4 below), the instructor needed to provide extra support to students in the form of virtual office hours, recorded lectures, and encouragement. Additional instructor support continued through 2021, as students navigated courses through a mix of in‐person and remote instruction due to the continuing COVID‐19 pandemic. Within the qualitative comments from 2020 and 2021 regarding student perception of their instructor, a common theme emerged in which students felt care, support, and encouragement. These comments were absent in other years of student evaluations. It is likely, then, that the support students felt during the challenging time of the COVID‐19 pandemic translated into a more positive course experience for these students.

## 4 CHALLENGES/REMOTE LEARNING/SOCIAL DISTANCING

One of the foremost challenges for F&F is the lack of an introductory‐level textbook that presents form and function in a manner that promotes synthesis of functional relationships across a diverse range of organisms. As a result, students must jump between a number of chapters or chapter sections in order to piece together the relevant information for understanding similarities and differences of a particular form and function relationship for several taxa (rather than proceeding through chapters in a relatively sequential manner). This can present challenges for students in navigating the textbook, leading to feelings of frustration and an impression that the textbook is useless (Box 2). In the future, we plan to explore creating custom online textbooks for the course using open‐access content such as those found on LibreTexts (LibreTexts, 2021).

Additional challenges arose in mid‐March 2020, when the COVID‐19 pandemic pushed many colleges and universities in the United States to shut down in‐person operations and move courses entirely online. Because F&F occurs in the second half of our college's spring semester, the entire F&F course was online. Students went home and had to juggle virtual courses with family obligations and differing time zones. To accommodate the needs of our students, we modified the course to be asynchronous yet still include the laboratory component. Video lectures were pre‐recorded and all quizzes, assignments, and tests were delivered and submitted electronically with Blackboard LMS. To modify the laboratory component for virtual, asynchronous delivery and to account for the lack of collaborative group work, weekly laboratories were reduced to the prelaboratory assignment and the station with pre‐recorded video data. Students still completed independent research, but were instead encouraged to select from a list of instructor‐provided hypotheses and use supplemental course videos for their analysis. To improve accessibility to students, the instructor provided additional weekend and evening virtual student hours to assist with experimental design and statistical analysis. Upon completion of their experiments, students submitted a final laboratory report and slideshow presentation for assessment.

Despite returns to in‐person instruction for the 2020–2021 academic year, social distancing requirements remained in place. As a result, course delivery methods for F&F needed to be reconsidered to accommodate the new requirements for in‐person instruction; a return to the prepandemic, in‐person course delivery method was not yet possible. Lectures were moved to a larger room on campus, which was retrofitted with appropriate technology to accommodate both in‐person teaching and live‐streaming for students joining class virtually (either as remote learners or due to quarantine/isolation requirements). Given the positive student feedback regarding some of the fully online F&F practices from spring 2020 (e.g., assignments; Box 2), we decided to maintain elements of the online course. For example, rather than offering individualized feedback on each homework submission, homework keys were posted after the assignment deadline. In addition, examinations continued to be administered via Blackboard LMS in spring 2021, but students took examinations while in class (in person). Doing so minimized paper consumption and risk of viral transmission between students and instructor, but also allowed real‐time interaction and problem‐solving with the professor if a student had a question or technical issue during the examination. As we return to more “normal” in‐person instruction in the 2021–2022 academic year, we anticipate keeping some elements from our pandemic teaching strategy. For example, we will continue to use homework keys instead of individual feedback on assignments, as this method provides students with a study tool to check their own work and significantly reduces turn‐around time on assignments (which is critically important for such a short‐term course). We also anticipate using some combination of online and paper examination formats moving forward. Opportunities for joining class remotely will likely not continue to be an option for courses; however, we plan to continue recording and posting lectures for students to review if they miss class due to illness or another conflict.

Laboratory space limitations necessitated a substantial restructuring of laboratory logistics for the 2021 offering of F&F. In order to accommodate distancing while still providing an in‐person laboratory experience for on‐site learners, we initiated laboratory rotations, during which half of the students in an assigned section attended laboratory in person for the first half of their scheduled laboratory time and the other half of the students attended in person for the second half of the laboratory period. However, this arrangement reduced the total amount of time that students were physically in laboratory, so we also revised the structure of the laboratory manual and laboratory activities to accommodate this change. In spring 2021, students worked through two manipulative stations while in the laboratory to collect data and collaborated with assigned laboratory partners outside of class to analyze data and complete the postlaboratory exercise. The remaining weekly laboratory station was reconfigured as a virtual prelaboratory exercise. Prelaboratories included nonexperimental activities aimed at developing students’ scientific research and writing skills (Table 2). Traditionally, laboratory assignments for F&F were submitted in hard copy; however, in spring 2021, we elected for digital submission using Google forms. This method provided an opportunity for students to complete assignments with their partners virtually using Google docs, thus eliminating the need for sustained close contact outside of class time. Additional adjustments to the laboratory were in place for spring 2021 to accommodate students approved for fully remote learning and for students who were temporarily unable to attend classes in person due to quarantine and isolation requirements. In these circumstances, students joined laboratory during their scheduled period via online video conferencing technology (e.g., Zoom; Google meet). The remote student “accompanied” their partner through the stations and participated virtually by engaging in conversation, asking questions, and serving as note‐taker and/or data recorder. The “in‐person” partner showed the remote student experimental materials and set up the screen so their partner could observe the manipulative work. This arrangement worked extremely well for most student groups. We anticipate returning laboratories to full capacity and eliminating the need to “split” sections as we return to nondistanced laboratory instruction in the 2021–2022 academic year. This will again allow students to have a full three‐hour in‐person laboratory experience. As such, we plan to return the remote prelaboratory assignments to in‐class activities for instructor‐guided scientific development. The use of Google docs to complete laboratory assignments promoted student collaboration in and outside of class; as a result, we anticipate keeping the online submission format for laboratory assignments moving forward.

## 5 CONCLUSION

Motivated by limitations of traditional biology survey courses, we developed a first‐year biology majors course aimed to help students gain a functional understanding of organismal biology through the lens of form and function. With our restructured course content, students explore similarities and differences in form among diverse taxa as they accomplish the functions of structure, organization, and movement; nutrients and feeding; transport and gas exchange; reproduction; and sensory systems. Furthermore, students explore these systems through instructor‐designed laboratory experiments and authentic research experiences. Student evaluations demonstrated that course organization strongly supported their learning progress. Once revised based on student feedback, assignments were also highly effective in helping students synthesize course concepts. Student perceptions of the course remained high even with modifications necessary during the COVID‐19 pandemic. We encourage other institutions to consider a form and function approach to teaching organismal biology and hope the material we have provided within this paper is helpful in inspiring course design.

## CONFLICT OF INTEREST

The authors declare no conflict of interest.

## AUTHOR CONTRIBUTION

**Laura N. Kloepper:** Conceptualization (equal); Data curation (equal); Formal analysis (lead); Writing‐original draft (equal); Writing‐review & editing (supporting). **Vanessa K Hilliard Young:** Conceptualization (equal); Data curation (equal); Formal analysis (supporting); Writing‐original draft (equal); Writing‐review & editing (lead).

## Data Availability

We provide summary data within the manuscript text. Selected course materials can be requested directly from the authors.
